# Knowledge, attitude and willingness to donate organ among medical students of Jimma University, Jimma Ethiopia: cross-sectional study

**DOI:** 10.1186/s12889-020-08931-y

**Published:** 2020-05-27

**Authors:** Fantu Kerga Dibaba, Kabaye Kumela Goro, Amare Desalegn Wolide, Fanta Gashe Fufa, Aster Wakjira Garedow, Birtukan Edilu Tufa, Eshetu Mulisa Bobasa

**Affiliations:** 1 Faculty of Health Sciences, School of Pharmacy, Jimma University, 378 Jimma, Ethiopia; 2 Facility of Medicine, Jimma University, 378 Jimma, Ethiopia; 3 Faculty of Health Sciences, School of Midwifery and Nursing, Jimma University, 378 Jimma, Ethiopia

**Keywords:** Organ donation, Knowledge, Attitude, Willingness, Medical students

## Abstract

**Background:**

The lack of organ donors has become a limiting factor for the development of organ transplantation programs. Many countries are currently facing a severe shortage of organs for transplantation. Medical students, as future doctors can engage in the role of promoting organ donation by creating awareness and motivating the community to donate their organs besides their voluntary organ donation. The aim of this study is to assess the knowledge, attitude and willingness of undergraduate medical students’ towards organ donation at Jimma University.

**Methods:**

A cross-sectional study was conducted among 320 medical students from year I to internship using questionnaire in order to assess their knowledge, attitude and willingness regarding organ donation. Data collected was entered using epidata and analyzed using Statistical Package for Social Sciences (SPSS) software version 20.

**Results:**

Mean (±SD = standard deviation) age of participants was 23.48 ± 17.025 years. 57.8% of the study subjects were male. There was a statistically significant interaction effect between gender and year of study on the combined knowledge questions (dependent variables) F(25,062) = 1.755,*P* = 0.014, Wilk’s *Λ* = .033. Variables which were related to a positive attitude towards organ donation were: being of the male sex (Odds Ratio = 1.156); having awareness about organ donation (Odds Ratio = 2.602); not having a belief on the importance of burying intact body (Odds Ratio = 5.434); willingness to donate blood (Odds Ratio = 4.813); and willingness to donate organ (Odds Ratio = 19.424).

**Conclusion:**

High level of knowledge but low level of positive attitude and willingness was noticed among the study participants toward organ donation.

## Background

The need for organ donation has increased globally in the past years due to an increase in organ failure [1]. Every day in the United States of America (USA), 21 people die waiting for an organ and more than 120,048 men, women, and children await life-saving organ transplants [2]. Accor-ding to a survey In India every year about 5 lakh (500,000) people die because of non-availability of organs and 1.5 lakh(150,000) people await a kidney transplant but only 5000 get among them [3]. Recently published report has found that approximately 3 million people in sub-Saharan Africa diagnosed with end-stage kidney disease (ESKD) die each year due to renal failure [4]. In Kenya, the kidney transplant queue at Kenyatta National Hospital in Nairobi stretches all the way to 2018, despite the hospital performing the procedure on a weekly basis [5]. In Ethiopia, between 130 and 150 corneas are collected yearly. However, there are more than 300,000 blind people waiting for corneal transplantation [6].

There are no sufficient facilities which provide maintenance and transplantation therapy for failed organs in Ethiopia. Currently there are only cornea and living related kidney transplant programs established in the nation’s capital Addis Ababa [6]. Facilities which provide maintenance dialysis has been in existence in the country starting from 2001. Hemodialysis has become on hand in private institutions, mostly in Addis Ababa the capital city of the country, and more recently in a few other urban and semi-urban regions. Currently, there are 30 hemodialysis centers with a total of 186 hemodialysis chairs and approximately 800 patients on hemodialysis. Among patients on maintenance dialysis, only about one-third receives treatment 3× per year because the cost of hemodialysis is unaffordable for the majority of patients [7].

Organ transplantation is one of the great advances in modern medicine and is the best option for failed organ. Transplantation is defined as the transfer of human cells, tissues or organs from a donor to recipient with an aim of restoring normal physiology in the body [8]. In Ethiopia, up to 2018, 1336 corneal and 90 living donor kidney transplants have been performed. Currently the kidney transplant program accepts candidates only at the age of 14 and above [7, 9].

Some studies found out that the issue of organ donation is multifactorial. In developed countries relational ties, religious beliefs, cultural influences, family influences, body integrity, and previous interactions with the health-care system were reported as the potential factors for organ donation [10]. However, there are limited studies regarding organ donation and the factors that influence it in developing countries for instance, in Kenya there are peoples who believe a person’s body should be intact when buried this belief and other sociocultural and legal factors hinder the harvest of organ from patients who have been medically declared to be in a “state of dying” [5].

Among 100,000 of people died each year are believed to be potential donors; however, only less than 200 actually become donors [11]. This indicates that a lot should be done on awareness creation towards organ donation. As a new approach in solving the organ shortage, it has been suggested that awareness about organ donation to be made a part of school education [12]. In Ethiopia we suggest to use religious leaders besides to incorporating the issue in school education, because Ethiopia is religious country. Our country has close ties with all three major Abrahamic religions, and it was the first in the region to officially adopt Christianity in the fourth century. Christians account for 63% of the country’s population, with 43.5% belonging to the Ethiopian Orthodox Church, 18.5% Protestant and 0.7% Catholic. Ethiopia has the first Hijra in Islamic history and the oldest Muslim settlement on the continent. Muslims account for 34% of the population, traditional 2.7% and other 0.6% [13].

In Ethiopia there are no data on public perception of organ donation and transplantation Therefore, the present study was designed to assess the knowledge, attitude and willingness of organ donation among medical students. Medical students, as future doctors can take up the role of promoting organ donation by educating and motivating the public to initiate them donate their organs besides their voluntary organ donation. Therefore, assessing medical student’s knowledge, attitude and willingness to donate organ is very important to decrease the shortage of organ in the future.

## Methods

### Study setting and subjects

A cross sectional study was carried out for 3 months from May to July 2019among under graduate medical students in Jimma University after obtaining Institutional Ethical Clearance from institutional review board (IRB) of Jimma University. The University is located in Jimma town which is 352 km from Addis Ababa, the capital city of Ethiopia. Jimma University is one of the most distinguished centers of excellence in medical education in the country.

### Sample size

All medical students (from first to internship) registered in the year 2018/2019 were the source population. Based on their training background, medical students in Jimma University were divided into two groups: PRE-CLINICAL and CLINICAL. PRECLINICAL is subdivided in to two groups: Year I (PC-I) and Year II (PC-II) and CLINICAL in to three subgroups Year III(C-I), Year IV(C-II) and internship. The sample size was calculated by using simple proportion formula assuming a prevalence of 50% for knowledge, attitudes and willingness of organ donation, a 95% confidence interval and a sample error of 5%. This was adjusted for 10% non-response rate; bringing the total sample size to 320.There were about 1200 students studying in Jimma University medical school.

### Study tool

The questionnaire was distributed to undergraduate medical students during lecture hours in the classroom and in ward during attachment. They were instructed not to discuss the questions among themselves. The importance of the study was explained and confidentiality regarding the participant response for the questions was ensured.

A 20-item self-administered questionnaire was developed. The first part of the questionnaire gathered the demographic details from the students, which included age, gender, year of study and religion. The second, third and fourth sections assessed the levels of knowledge (Q1–7), attitude (Q8–16) and willingness (Q17–20) to donate organ, respectively.

The students were grouped as those who do have adequate and inadequate knowledge based on their score.

**Adequate knowledge** is when 4–6 questions were answered correctly and inadequate when less than 4 questions answered correctly out of 6 knowledge questions.

Attitude was assessed by using 9 attitude statements and respondents were categorized as those who do have positive attitude and negative if they agree to 6–9 and less than 6 attitude statements respectively.

### Statistical analysis

Data was entered to EPI data and exported to SPSS version 20 for analysis. Descriptive statistics like percentage and mean and standard deviation were used to present socio-demography, knowledge, attitude and willingness response of the participants. Multivariate analysis was used in order to relate those factors that gave a significant result: One way Multivariate analysis of variance (MANOVA) was used to see a significant relationship between one independent variable and dependent variables and two ways MANOVA was considered to know if there was an interaction between two independent variables on the dependent variables. One way Analysis of Variance (ANOVA) was used for comparing means of variables to know among which groups were the differences. Finally, Odds ratio analysis was used to find out variables which were related to a positive attitude towards organ donation.

## Result

Out of 320 participants 57.8% were male. Mean (±SD = standard deviation) age of participants was 23.48 ± 17.025 years. Majority of the participants were orthodox (49%.7) and the least percentage being others constituting wakeefeta, apostolic, humanity, atheist and Seventh Day Adventist (SDA) (2.8%) (Table 1).

**Table 1:** Sociodemographic data of the participants

Sociodemographic variables	*N* = 320(%)
**Age**	
18–19	33 (10.3)
20–21	121 (37.8)
22–23	87 (27.2)
24–25	66 (20.6)
> 25	13 (4.1)
Mean age ± SD = 23.48 ± 17.025	
**Sex**	
Male	185 (57.8)
Female	135 (42.2)
**Religion**	
Orthodox	159 (49.7)
Protestant	109 (34.1)
Muslim	43 (13.4)
Others	9 (2.8)
**Year of study**	
PC-I	46 (14.4)
PC-II	60 (18.8)
C-I	66 (20.6)
C-II	87 (27.2)
Internship	61 (19.1)

96.9% of the students had awareness about organ donation. Only 25% had knowledge that there was no age limit for organ donation (Table 2).

**Table 2:** Knowledge of medical students regarding organ donation (*n* = 320)

S. no	Question	No of students answered correctly (%)
1	Have you ever heard about Organ Donation? (Yes/No)	310 (96.9)
2	The term ‘Organ Donation’ means?	237 (74.1)^a^
3	Do you know the meaning of brain death? (Yes/No)	282 (88.1)
4	Which organs can be donated?	206 (64.4)^b^
5	Is there age limit for donating organs? (Yes/No)	80 (25)
6	All religions support organ donation (True/False)	274 (85.6)

^a^Medical students who had chosen the correct definition of ‘Organ Donation’; ^b^Medical students who had correctly identified all the given organs that can be donated

There was a statistically significant difference in level of knowledge between study groups as demonstrated by one-way ANOVA(F (4,315) =7.6, *p* = 0.001). Based on the post hoc test the significant difference was between PC-I and C-II(*p* = 0.001), PC-I and intern(*p* = 0.001), PC-II and C-I(*P* = 0.022) and PC-II and intern(*p* = 0.010). The mean for PC-I, PC-II, C-I, C-II and intern is 1.37, 1.27, 1.20, 1.08 and 1.05 respectively. Therefore, PC=I had significantly higher level of knowledge when compared to the rest year of study (Table 3).

**Table 3:** Comparison of level of knowledge of study participants based year of study

Year of study	Year of study	*P*
PC-I	PC-II	.606
	C-I	.104
	C-II	.001*
	Intern	.001*
PC-II	C-I	.823
	C-II	.022*
	Intern	.010*
C-I	C-II	.293
	Intern	.156
C-II	Intern	.986

*The mean difference is significant at 0.05 level

74.1% of the participants agreed to support family members if they wish to become an organ donor. Majority of the study subjects (91.9%) felt that awareness about organ donation should be made a part of school education (Table 4).

**Table 4:** Attitude of medical students regarding organ donation (*n* = 320)

S. No	Attitude statements	Subjects who replied yes(%)
1	Would you like to donate your organs?	216 (67.5)
2	Do you feel comfortable to think or talk about organ donation?	256 (80)
3	Would you like to motivate others to donate organs?	251 (78.4)
4	Do you support your family members if they wish to become an organ donor?	237 (74.1)
5	Do you think awareness about organ donation should be made a part of school education	294 (91.9)
6	It is important for a person’s body to have all of its parts when buried	77 (24.1)
7	Would you like to donate your organ to anybody?	177 (55.3)
8	Do you think donating an organ can cause any harmful effects / complication to you?	186 (58.1)
9	Do you believe that there is a danger that donated organs could be misused, abused or misappropriated?	274 (85.6)

According to our finding, males were 1.156 (Odds Ratio = 1.156) times likely to have positive attitude towards to organ donation as compared to female. Students who had an awareness about organ donation were 2.602 (Odds Ratio = 2.602) times likely to have positive attitude towards to organ donation as compared to those who were unaware. The other variables which were related to a positive attitude towards organ donation were: not having a belief on the importance of burying intact body (Odds Ratio = 5.434); knowing definition of brain death (Odds Ratio = 1.257); not having a belief that there is a danger of misuse, abuse or misappropriation of donated organ (Odds Ratio = 2.777); willingness to donate blood (Odds Ratio = 4.813); and willingness to donate organ (Odds Ratio = 19.424).

**Fig. 1:**
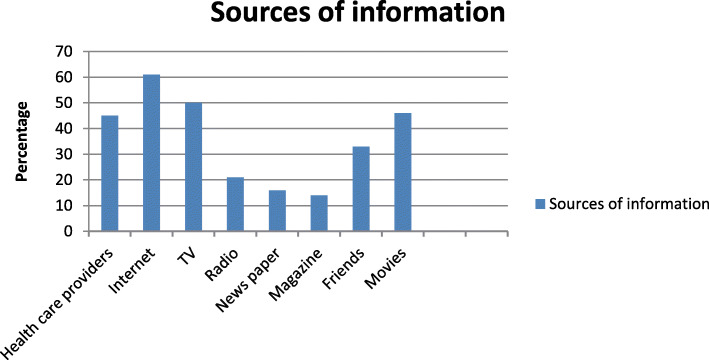
Distribution of study subjects according to the source of information about organ donations. i.e. Note: No of respondents may be greater than sample size as multiple options were allowed. Most common source of information about organ donation was found to be internet (61%) television (50%) followed by, Movies and health care providers 46 and 45% respectively

58.1% of the study participants were willing to donate their organs and allow organ donation after the death of a family member. Majority of the study subjects (88.4%) did not like to take money for organ donation. 90.3% of the study subjects were willing to donate blood and 58.1% were willing to donate their organ (Table 5) (Fig. 1).

**Table 5:** Willingness among medical interns regarding organ donation (*n* = 160)

S. no	Questions	Students who replied yes (%)
1	Are you willing to donate blood?	289 (90.3)
2	Are you willing to donate your organ?	186 (58.1)
3	Are you willing to allow organ donation after the death of a family member?	186 (58.1)
4	Would you like to take money for organ donation?	37 (11.6)

There were an association between willingness and attitude. Willingness to donate organ was significantly higher among those who do have positive attitude (88.2%) as compared to those with negative attitude (11.8%) (Table 6).

**Table 6:** Association of attitude and willingness to donate organ

Are you willing to donate your organ?	Attitude		Total
	Positive(%)	Negative(%)	
Yes	164 (88.2)	22 (11.8)	186 (100)
No	37 (27.6)	97 (72.4)	134 (100)
Total	201 (62.8)	119 (37.2%)	320 (100)

χ2 = 122.3; df = 1;*P* < 0.001

There was a statistically significant difference on belief of burying intact body between religions as demonstrated by one-way ANOVA(F (3,316) =4.5, *p* = .004). Based on the post hoc test the significant difference was between Protestant and Muslim (*p* = .007). The mean for protestant is 1.83 and Muslim 1.56.Therefore, Protestant had significantly higher belief on the importance of burying intact body when compared to Muslim (Table 7).

**Table 7:** Comparison of belief of participants on the importance of burying intact body based on religion

Religion of the subject	Religion of the subject	*P*
Orthodox	Protestant	.677
	Muslim	.051
	Other	.853
Protestant	Muslim	.007*
	Other	.980
Muslim	Other	.207

*The mean difference is significant at 0.05 level

There was a statistically significant difference between males and females when knowledge questions considered jointly Wilk’s *Λ* = .96, F (6,312) = 2.247, *P* = 0.039, multivariate ƞ^2^ = 0.041 and attitude statements consider jointly Wilk’s *Λ* = .94, F (9,310) = 2.301, *P* = 0.016, multivariate ƞ^2^ = 0.063.

When year of study is considered, there was a statistically significant difference among year of studies when knowledge questions considered jointly Wilk’s *Λ* = .75, F (25,079) = 3.966, *P* < 0.001, multivariate ƞ^2^ = .071, attitude statements considered jointly Wilk’s *Λ* = .77, F (37,152) = .766, *P* < 0.001, multivariate ƞ^2^ = .065 and willingness questions considered jointly Wilk’s *Λ* = .93, F (12,828) = 2.072, *P* = 0.017, multivariate ƞ^2^ = .026.

Two way MANOVA was considered to know if there was an interaction between two independent variables on the dependent variables. There was a statistically significant interaction effect between gender and year of study on the combined knowledge questions (dependent variables) F (25,062) = 1.755, *P* = 0.014, Wilk’s *Λ* = .033.

## Discussion

### Knowledge of the participant

Organ failure and shortage of donated organs are global problem. Among 100,000 of people died each year are believed to be potential donors; however, only less than 200 actually become donors [9]. The widespread shortage of donated organs indicates that there is low donor rate worldwide; In Ethiopia there is no data on rate of organ donation. In 2017 Spain had the highest donor rate in the world at 46.9 per million people, followed by Portugal (34.0 per million), Belgium (33.6 per million), Croatia (33.0 per million) and the US (32.0 per million) [14]. Donated organs are the major pre-requisite for consistency of organ transplantation program; one of the solutions to increase organ supply is to assess public knowledge, attitude and willingness towards organ donation and taking an action based on the data. In our country there is no study done on people’s perception towards organ donation this background pledges us to conduct this study.

In our study 96.9% of the participants heard about organ donation which is similar to study done by Annadurai et al and Jothula et al. [15, 16] both reported that 100% of the participants were aware about organ donation.74.1% of the participants were aware about the meaning of organ donation which is relatively higher than the study done by Annadurai et al. [15]. In the present study, level of knowledge was significantly higher among PC=I (year I) students as compared to the other year of study this finding was similar to study done among undergraduate dental students of Panineeya Institute of Dental Sciences and Hospital, which showed higher average knowledge among first-year students [17]. In this study, only 82.5%of medical students had adequate knowledge about organ donation which is relatively higher than the study done on final semester medical students by Karini et al. which showed that only 56% of them were having adequate knowledge [18].

In the present study the main sources of information about organ donation was found to be internet (61%) and television (50%).This was similar to study conducted in USA and Australia [19, 20]. However; Similar findings were observed by Sindhu et al. and Jothula KY et al. [16, 21]. The third source of information about organ donation in our study are health care providers (45%) which is relatively higher than the study done by Annadurai et al. [15] which reported 34.1%. this finding showed that health care providers are playing undeniable role in creating awareness towards organ donation in Ethiopia.

206(64.4%) of our study participants had identified all the organs that can be donated. This finding was higher than the study done by Annadurai et al. [15] and Karini et al. [18] which reported 16.1 and 26% respectively. In the present study 80(25%) of the students knew that there is no age limit for organ donation which is approximate to Sucharitha et al. and lower than Jothula KY et al. [16, 22].

### Attitude of medical students regarding organ donation

201(62.8%) of our study subjects have a positive attitude towards organ donation which is lower than the study in Spain and India which found 80 and 71.3% respectively [23, 24]. 91.9% of this study subjects, felt that awareness about organ donation should be included in school curriculum which is similar to Adithyan et al. reported that 91.2% of the subjects felt the need for revision of medical curriculum on organ donation [25] Our study found out that 251(78.4%) of the study subjects would like to motivate others for organ donation which is lower than to the Vinay et al [26].

77(24.1%) of our study subjects belief that person’s body should be intact when buried A study in USA reported that 8% of participants strongly agree and 11.7% agree to this statement which is almost similar to our finding [19]. In our study being of the male sex (Odds Ratio = 1.156) was related to a favorable attitude towards to organ donation; in contrast, a study done in Spain reported that being of females sex (Odds Ratio = 1.739) was related to a favorable attitude [23]. In our study not having a belief on the importance of burying intact body (Odds Ratio = Ratio = 5.434) was one of the variables which affect positive attitude towards to organ donation which was similar to a study in USA [19]. A study done in Spain reported being a blood donor (OR = 2.824) as a variable related to a positive attitude towards to organ donation similarly in our study we found out willingness to donate blood (Odds Ratio = 4.813) as a variable to a favorable attitude.

### Willingness of medical students to donate organ

In this study 186(58.1%) of the study participants were willing to donate their organ which is similar to a study done in USA [20] and lower than Payghan et al. and Vinay et al revealed that almost 90% of study participants were willing to donate their organs [26, 27]. The present study found out that there is a significant association between attitude regarding organ donation and willingness to donate organs which is different from the finding by Ali et al. and by Dasgupta et al. [28, 29] which reported that there was a significant association between attitude and knowledge acquired. Though taking money for organ donation is unethical 11.6% of our study participants would like to take money for organ donation which was higher than study by Jothula KY et al. [16].

## Conclusion

Though most of the students had adequate knowledge, still gaps exist in their attitude and willingness. This implies the need for an intensified and sustained education to raise attitude and willingness of the students towards organ donation.

## Recommendations

Most of the students (91.9%) felt that awareness about organ donation should be made a part of school education; until it included in school curriculum, we recommend the students to acquire an adequate knowledge by themselves; In our study the most common source of information about organ donation was internet; so, they can browse more to acquire additional knowledge and make informed decision.

## Data Availability

The datasets used and/or analysed during the current study are available from the corresponding author on reasonable request.

## Abbreviations

ANOVA: Analysis of variance
C-I: Clinical-I
C-II: Clinical-II
ESKD: End-stage kidney disease
IRB: Institutional Review Board
JUMC: Jimma University Medical College
MANOVA: Multivariate analysis of variance
PC-I: Pre-clinical-I
PC-II: Pre-clinical-II
SDA: Seventh Day Adventist
SPSS: Statistical Package for Social Sciences
USA: United States of America
